# Processed pseudogene insertion in *GLB1* causes Morquio B disease by altering intronic splicing regulatory landscape

**DOI:** 10.1038/s41525-022-00315-y

**Published:** 2022-07-26

**Authors:** Igor Bychkov, Antonina Kuznetsova, Galina Baydakova, Leonid Gorobets, Vladimir Kenis, Alena Dimitrieva, Alexandra Filatova, Vyacheslav Tabakov, Mikhail Skoblov, Ekaterina Zakharova

**Affiliations:** 1 Research Centre for Medical Genetics, Moscow, Russia; 2 Clinical and Diagnostic Center “Zdorovoe detstvo”, Rostov-on-Don, Russia; 3 H. Turner National Medical Research Centre for Children’s Orthopedics and Trauma Surgery, Saint Petersburg, Russia

**Keywords:** Metabolic disorders, Mobile elements, RNA splicing

## Abstract

Morquio B disease (MBD) is an ultra-rare lysosomal storage disease, which represents the relatively mild form of *GLB1*-associated disorders. In this article, we present the unique case of “pure” MBD associated with an insertion of the mobile genetic element from the class of retrotransposons. Using whole-genome sequencing (WGS), we identified an integration of the processed pseudogene *NPM1* deep in the intron 5 of *GLB1*. The patient’s mRNA analysis and the detailed functional analysis revealed the underlying molecular genetic mechanism of pathogenesis, which is an alteration of the *GLB1* normal splicing. By co-expression of minigenes and antisense splice-modulating oligonucleotides (ASMOs), we demonstrated that pseudogene-derived splicing regulatory motifs contributed to an activation of the cryptic exon located 36 bp upstream of the integration site. Blocking the cryptic exon with ASMOs incorporated in the modified U7 small nuclear RNA (modU7snRNA) almost completely restored the wild-type splicing in the model cell line, that could be further extended toward the personalized genetic therapy. To our knowledge, this is the second reported case of the processed pseudogene insertion for monogenic disorders. Our data emphasizes the unique role of WGS in identification of such rare and probably underrepresented in literature types of disease-associated genetic variants.

## Introduction

Morquio B disease (MBD, MIM: 253010) or Mucopolysaccharidosis IVB is a lysosomal storage disorder associated with pathogenic variants in the *GLB1* gene (NM_000404.4). Alternatively spliced mRNA isoforms of this gene encode the enzyme β-galactosidase (β-GAL) and the elastin binding protein^1^. The mature β-GAL is located in lysosomes and is involved in degradation of gangliosides, glycoproteins, and glycosaminoglycans^2^. Various combinations of pathogenic variants in *GLB1* have different effects on the β-GAL activity toward its main substrates—GM1 ganglioside and keratan sulfate, which further gives a rise to the continuum of phenotypes. The most severe form of the disease is the infantile-onset GM1 gangliosidosis, caused by massive accumulation of the GM1 ganglioside and related glycoconjugates in the central nervous system with subsequent progressive neurodegeneration. On the other side of the phenotypic continuum, lies MBD, which is mainly associated with impaired degradation of keratan sulfate and its accumulation in skeletal tissue.

Clinically, MBD represents a relatively mild form of Morquio A disease, caused by the deficiency of another lysosomal enzyme—galactosamine 6-sulfatase (*GALNS*). The “pure MBD” manifests with progressive growth impairment and characteristic dysostosis multiplex, which generally includes three or more radiological/clinical findings: platyspondyly and vertebral beaking involving all segments of the spine, odontoid hypoplasia, epi- and metaphyseal dysplasia of long bones, genua/coxa valga, hip dysplasia, joint laxity/hyperextensible joints, barrel chest/pectus carinatum, and short stature^3,4^. Neuronopathic features can be presented in a small proportion of MBD patients, which can be classified by the “MBD plus” phenotype. The laboratory diagnostics of MBD is based on measurement of the β-GAL activity in blood cells and detection of keratan sulfate in plasma and urine by the Liquid Chromatography with Tandem Mass Spectrometry (LC-MS/MS) method, followed by identification of biallelic variants in the *GLB1* gene. The number of therapies for *GLB1*-associated disorders is currently developing, including AAV9-mediated gene therapy^5^, enzyme replacement therapy^6^, substrate reduction therapy^7^, and chaperone-based therapy^8^.

MBD is an ultra-rare disease with an estimated prevalence of 1:250,000 to 1:1,000,000 live births and about 62 published cases^4^. To date, 25 disease-causing mutations have been described for *GLB1* in the Human Gene Mutation Database (http://www.hgmd.cf.ac.uk/ac, accessed on 5th Jan 2022), most of which (88%) are missense variants. The small number of reported patients hinders the analysis of genotype-phenotype correlations, although there are two common for MBD variants: c.817_818delinsCT (p.Trp273Leu) and c.1498A>G (p.Thr500Ala). The c.817_818delinsCT variant is invariantly associated with the “pure MBD” as it mainly affects the keratan sulfate degradation^9^.

In this article, we present the unique case of the “pure” MBD associated with an insertion of the processed pseudogene (PP) *NPM1* deep in the intron 5 of *GLB1*, identified by WGS. PP is a mobile genetic element (MGE) from the class of retrotransposons. MGEs comprise more than two-thirds of the human genome and provided it with a large variety of functionally significant sequences, including promoters, enhancers, transcription terminators, small RNA genes, and those shaping the chromatin structure^10–12^. Depending on the intermediate molecule participating in transposition, MGEs are divided into two major classes—DNA transposons and RNA or retrotransposons^13,14^. If the MGE encodes all of the necessary for its own transposition elements, it is called autonomous. The most abundant autonomous transposons in the human genome are long interspersed elements (LINEs). LINE encodes ORF1 and ORF2 proteins, which incorporate the LINE’s RNA or the RNA of nonautonomous retrotransposons and after reverse transcription integrate it into the genomic locus. In some rare cases, these proteins can mobilize the mRNA of protein-coding genes with generation of PP insertions^15^. The human genome contains more than 10,661 PPs (https://www.gencodegenes.org/human/stats.html, accessed on 9th Jun 2022), most of which are ribosomal protein genes^16^.

Active MGEs are involved in such physiological processes as normal brain development, but, on the other hand, contribute greatly to human genetic diseases and cancer^17–20^. The molecular mechanisms of pathogenesis, by which MGEs alters genes’ expression, include transposition-associated deletions, disruption of the gene’s coding sequence, change of methylation and splicing patterns, and premature transcription termination^19–21^. For example, the only previously reported PP insertion, associated with human monogenic disease, caused the alteration of the *CYBB* gene splicing by inclusion of the cryptic exon^22^.

Herein, we report the second case of such insertion and demonstrate that the identified PP in *GLB1* also altered the host’s gene splicing but due to the more complex molecular mechanism and can be a potential susceptible target for splice-modulating therapy.

## Patient’s summary

**Fig. 1:**
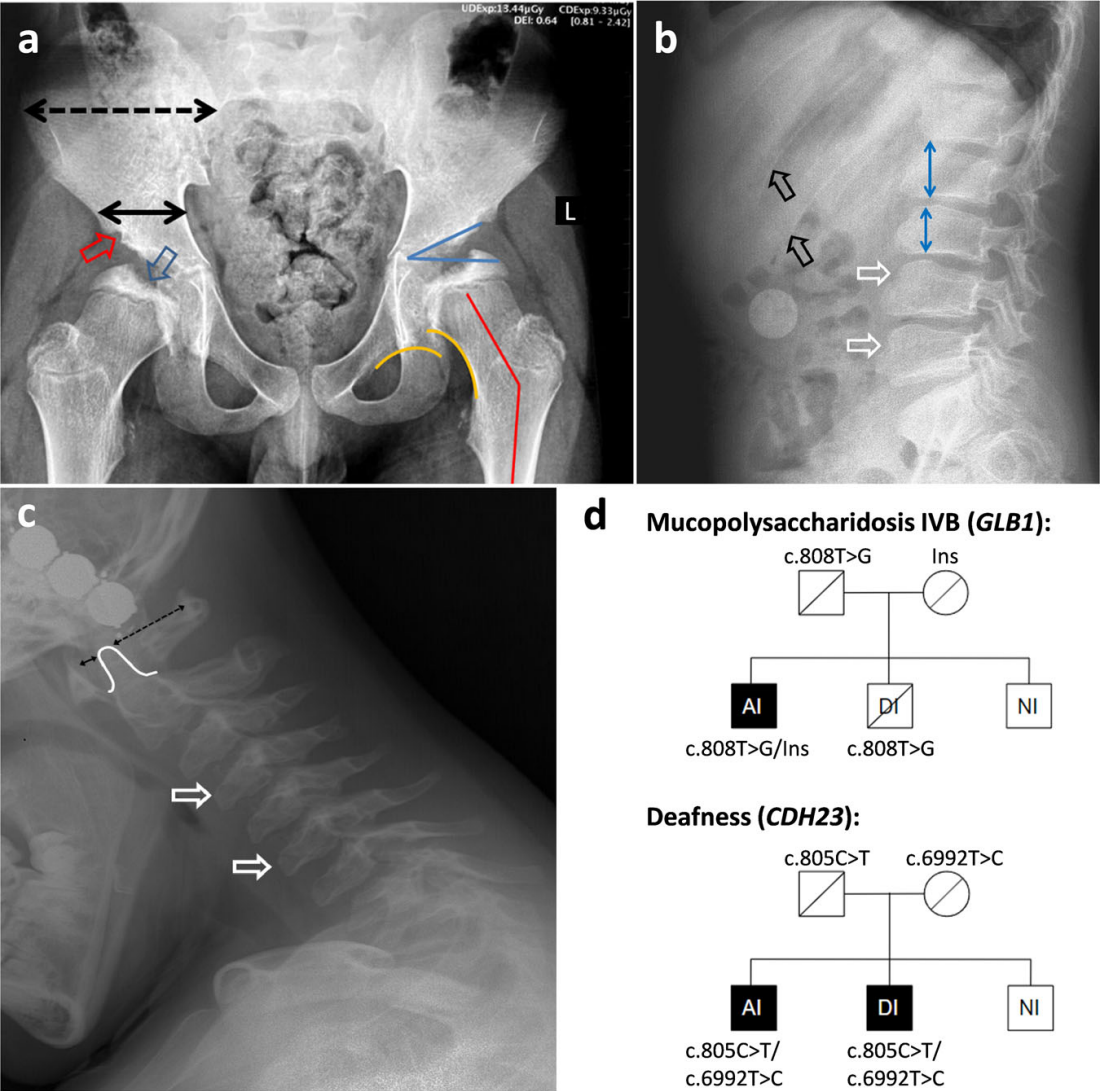
Patient’s summary. **a** The anteroposterior radiograph of pelvis and hips: “mickey mouse” pelvis—wide iliac wing (dotted black line) and narrow iliac body (solid black line); deficient ossification of acetabular edge (red arrow) and femoral head (blue arrow); dysplastic acetabulum (acetabular angle 30°—blue lines); coxa valga (neck-shaft angle 150°—red lines); disrupted Shenton line (yellow lines). **b** Radiograph of the lumbar spine (lateral view in flexion): anterior wedging and beaking of the lumbar vertebrae (white arrows); anisospondyly—irregular height of the vertebral bodies in cranio-caudal direction (blue lines); widening of the ribs (black arrows). **c** Radiograph of the cervical spine (lateral view): anterior wedging and beaking of the cervical vertebrae (white arrows); mild hypoplasia of the C2 dens (white line); mild anterior atlanto-axial instability (black lines). **d** Segregation analysis of the variants, associated with MPS IVB and nonsyndromic deafness in the patient’s family. Ins—insertion of the processed pseudogene. Painted icons—affected patients, crossed icons—heterozygous asymptomatic carriers.

Patient AI is a 9-year-old boy who was admitted to an orthopedic outpatient clinic due to the gait disturbance and pain in the right hip region. The gait disturbance appeared at 6 years and 10 months and was associated with an acute upper respiratory tract infection episode. The patient was under supervision of a pediatrician but the symptoms slowly progressed. The radiological examination in the orthopedic clinic revealed specific features of the dysostosis multiplex: bilateral lesions of the femoral heads, dysplastic acetabulum, platyspondyly with ventral wedging of the cervical vertebrae, and anisospondyly with tongue-shaped beaking of the lumbar vertebrae (Fig. 1a–c).

Patient AI was also diagnosed with sensorineural hearing loss of 4th degree in early childhood. The patient has two older brothers: one (DI, 11-year-old) has the sensorineural hearing loss of 4th degree and the other (NI, 17-year-old), who has neither hearing impairment nor any complaints from the musculoskeletal system. Both AI and DI underwent cochlear implantation at the age of 1 and 9 years, respectively.

Based on the characteristic radiological signs, a disease from the group of lysosomal storage disorders was suspected. The subsequent biochemical analysis of multiple lysosomal enzymes in dried blood spots revealed a significant decrease of the β-GAL activity in patient AI (0.72 and 0.4 nM/ml/h with normal range: 2–30 nM/ml/h), which is the marker of *GLB1*-associated disorders.

## Results and discussion

### Identification of the causative variants

Analysis of the *GLB1* gene for patient AI was initially performed by Sanger sequencing followed by whole-exome sequencing. Both methods identified only one pathogenic variant c.808T>G (p.Tyr270Asp) in *GLB1* in heterozygous state. The variant was also identified in heterozygous state in the patient’s father and brother DI (Fig. 1d).

As patient AI and his older brother DI both have severe hearing impairment, but DI did not show any signs of MBD, we hypothesized whether there is a separate genetic cause responsible for the non-syndromic deafness. We analyzed whole-exome sequencing data for the variants in genes associated with non-syndromic deafness and identified two likely pathogenic compound heterozygous variants c.805C>T (p.Arg269Trp) and c.6992T>C (p.Val2331Ala) in the *CDH23* gene (additional information is in the Supplementary Note 1).

To identify the second causative variant in *GLB1* responsible for the recessive phenotype of MBD, we performed WGS. In addition to the c.808T>G variant, identified earlier, the structural variant caller (Manta) detected DNA break ends in the intron 5 of *GLB1* and the group of discordant reads, whose mates were mapped to the *NPM1* gene. Furthermore, the analysis of reads using the Integrative Genomics Viewer (IGV) revealed that discordant reads in *GLB1* span a duplication of 16 bp (NC_000003.11:g.33100046_33100061). The duplication “AAAGTATCTACTTTCT” (relative to the sense strand) overlaps with the recognition site of the retroviral integrase “TTTAAAGTA”^23^. Such duplications are formed during the target-primed reverse transcription of the retroviral RNA and are hallmarks of mobile genetic elements insertion from the class of retrotransposons^24^. The subsequent analysis of discordant reads in *GLB1* using the IGV identified that their mates were mapped to the first and the 11-th (last) exon of *NPM1*. Since retrotransposons use the RNA molecule as an intermediate, an integration of the *NPM1* coding sequence was suspected. This type of retrotransposition occurs when retroviral proteins incorporate the mRNA of protein-coding genes and after reverse transcription integrate it into the DNA locus^15^.

Amplification of the insertion breakpoint by PCR identified an additional high molecular band in patient AI and his mother. Sanger sequencing of this band revealed, that, indeed, the insertion represents 1301 bp of the *NPM1* cDNA (corresponding to c.-97_ *319), flanked by the single guanine at the 5′ end, acquired during capping, and polyA tail at the 3′ end. Thus, the insertion of the PP *NPM1* in the intron 5 of *GLB1* was established in a trans-position with the missense c.808T>G variant (Fig. 1d).

### Patient’s RNA analysis

**Fig. 2:**
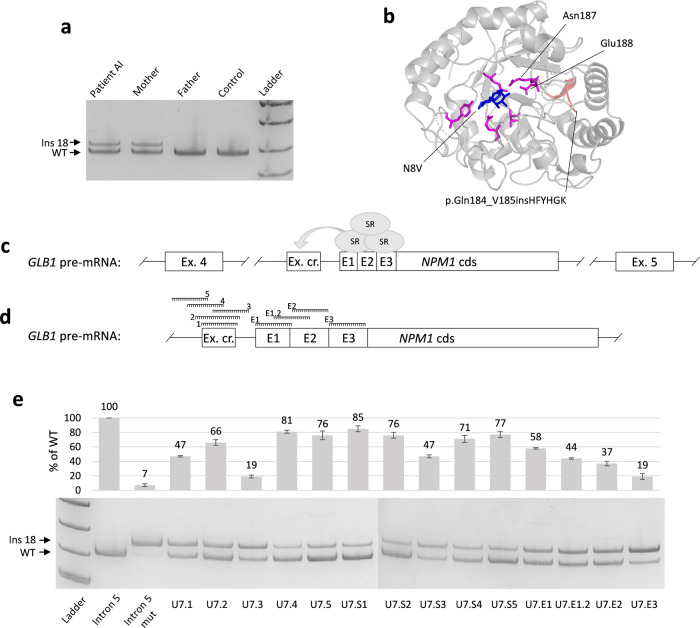
Results of functional analysis of the PP insertion. **a** Results of the blood mRNA analysis demonstrated the presence of an additional *GLB1* mRNA isoform with inclusion of the 18 bp fragment of the *GLB1* intron 5 in patient and his mother, which is the heterozygous carrier of the PP insertion at the DNA level. **b** Fragment of the patient’s mutated β-GAL with the insertion of six amino acids (the 3D model was created by SWISS-MODEL based on the PDB:3WEZ template), located in the close proximity to the protein’s active site and two amino acids upstream of the catalytic residue Asn187. Catalytic residues are colored in pink, insertion is red and N8V ligand is blue. **c** The scheme of the *GLB1* intron 5 fragment at the pre-mRNA level. The cryptic 18 bp exon (Ex. cr.) is located 36 bp upstream of the PP insertion (*NPM1* cds) and was supposed to be activated by the PP-derived splicing enhancer motifs (E1-3). Splicing enhancers are enriched in binding sites of serine-rich proteins (SR), which promote the inclusion of the exons by interacting with spliceosome. **d** Location of studied ASMOs. Three clusters of splicing enhancer motifs were predicted by HExoSplice and were chosen as targets for ASMOs together with the cryptic exon (additional information is in Supplementary Figs. 1 and 2). **e** Fragment analysis and representative polyacrylamide gel electrophoresis visualization of PCR products obtained from minigene experiments. HEK293T cells were transfected with minigenes containing the fragment of the WT intron 5 and the mutated one (with PP insertion) alone (columns 2 and 3) or co-transfected with the mutated minigene and plasmids expressing modU7snRNAs (columns 4–17). Columns 4–8 (U7.1-5) correspond to modU7snRNAs targeting cryptic exon. Columns 9–13 (U7.S1–S5) correspond to the same modU7snRNAs but tailed with the splicing silencer hnRNPA1 motif. Columns 14–17 (U7.E1-E3) correspond to the modU7snRNAs targeting PP-derived splicing enhancer motifs. Error bars represent the standard deviation of biological replicates. The uncropped blots are presented in Supplementary Fig. 3. All blots derive from the same experiment and were processed in parallel.

To identify possible splicing alterations, caused by the PP insertion analysis of the *GLB1* mRNA obtained from white blood cells was performed. The *GLB1* cDNA amplification revealed an additional product in the patient AI and his mother, which turned out to be an insertion of the 18 bp fragment of the intron 5 (NC_000003.11:g.33100082_33100099) between exons 5 and 6 (r.552_553insCATTTCTACCATGGGAAG) (Fig. 2a). At the DNA level, the inserted sequence is located 36 bp upstream of the PP integration site and represents a cryptic exon (Fig. 2c). This cryptic exon has reliable acceptor and donor splice sites (MaxEnt score 9.97 and 7.04 respectively) but is located in the region, highly enriched with splicing silencers’ motifs, that suppresses the exon recognition and overall splicing process in the vicinity (Supplementary Fig. 1). The PP sequence on the other hand is enriched with splicing enhancer motifs, as it consists of exons. Thus, we hypothesized, that the altered landscape of splicing regulatory motifs in intron 5 led to activation of the cryptic exon and insertion of the 18 bp cryptic exon in the mature *GLB1* mRNA.

At the protein level, this insertion (p.Gln184_V185insHFYHGK) affects the highly conservative beta-strand (amino acids 177–189) in the TIM barrel domain (Fig. 2b). This strand contains two catalytic residues Asn187 and Glu188 in close proximity downstream of the inserted amino acids^25^. Several bioinformatics algorithms also predicted the highly deleterious effect of the insertion (Supplementary Note 2). Thus, the insertion, most probably, severely alters the active site configuration and enzyme activity.

### Study of the molecular mechanism of pathogenesis of the processed pseudogene insertion

To confirm that the PP insertion caused an activation of the cryptic exon, we created two expression vectors or “minigenes”, in which the wild-type (WT) and the mutated (with PP insertion) fragments of the *GLB1* intron 5 were placed between two constitutively spliced exons. Minigenes were transfected into HEK293T cells and after 48 h, mRNA was extracted and analyzed for the minigene-specific splicing outcome. The results of minigene assay demonstrated similar to the patient’s *GLB1* cDNA splicing pattern—insertion of the 18 bp cryptic exon in the vast majority of mRNA molecules (Fig. 2e—columns 2 and 3). In addition, some residual amount of the WT isoform was detected (7%), which suggests that this allele is “leaky” and probably hypomorphic. After the confirmation of the deleterious effect of the PP insertion on the gene’s splicing, we classified it according to the ACMG guidelines^26^ as the likely pathogenic variant (PM2 moderate, PM3 moderate, PM4 moderate, PP4 supporting).

To prove that PP-derived splicing enhancers caused an activation of the cryptic exon and to develop an approach to the personalized genetic therapy for this variant, we designed an experiment based on the co-transfection of minigenes and antisense splice modulating oligonucleotides (ASMOs) in the HEK293T cell line. Using HExoSplice, we identified three regions at the 5′ end of the insertion, with the highest density of splicing enhancer motifs and designed the corresponding antisense sequences (Fig. 2d and Supplementary Figs. 1 and 2). ASMOs targeting splicing enhancers located in the PP insertion and the cryptic exon were incorporated into modified U7 small nuclear RNA (modU7snRNA) genes, which were cloned into expression vectors.

The results of the co-transfection experiments demonstrated that all of modU7snRNAs significantly (*p* < 0.01 by unpaired *t* test) restored the WT splicing to some extent (Fig. 2e). ASMOs targeting PP-derived splicing enhancers (Fig. 2e—U7.E1-3) inhibited the inclusion of the cryptic exon in a position-dependent manner. Blocking of the proximal enhancer (E1 at Fig. 2d) led up to 58% of the WT isoform recovery. Blocking of the more distal splicing enhancers led to 37% and 19% of WT isoform respectively, which suggests that at least 123 bp of the PP-derived sequence contributed functionally to the cryptic exon activation.

ASMOs targeting the cryptic exon demonstrated the highest efficiency when being shifted to the acceptor splice site (Fig. 2e—81% for U7.4 and 76% for U7.5). An addition of motifs of the splicing silencer hnRNPA1 to an antisense sequence (U7.S1–S5) improves the efficiency for all of modU7snRNAs except U7.4. This observation may be explained by the fact that exonic splicing enhancers and silencers can both improve or inhibit inclusion of the exon, depending on their relative position^27^.

The efficient restoration of the patient’s *GLB1* WT splicing by modU7snRNAs can be further extended toward the personalized genetic therapy based on AAV9 vectors, as the modU7snRNA cassette is about 500 bp in length and can be easily incorporated into any AAV particles. The treatment of rats with Morquio A disease by AAV9 vectors containing the *GALNS* gene demonstrated the widespread transduction of bones, cartilage, and peripheral tissues and reduction of keratan sulfate levels^28^. Thus, this type of delivery system can be also effectively tested for MBD animal models and can be used for delivering patient-specific ASMOs.

### Genotype–phenotype correlations

The identified c.808T>G missense variant in *GLB1* is a severe variant, located in the protein’s active site and leading to the complete absence of enzymatic activity, when being expressed in COS-1 cells^29^. c.808T>G is associated mainly with infantile-onset form of the GM1 gangliosidosis and supposed to be a common pathogenic variant in *GLB1*.

The second identified likely pathogenic allele in *GLB1* represents the PP insertion, which leads to an inclusion of the cryptic exon in the mature mRNA. At the protein level, the resulting insertion affects the highly conservative beta-strand containing catalytic residues and probably severely alters the activity of β-GAL. Taking into account the presence of the highly deleterious variant c.808T>G in one allele, we suggest, that the reason for the relatively mild phenotype of our patient is the significant residual amount of WT mRNA isoforms produced by the PP-containing alelle.

There is one reported case, where c.808T>G was found in compound-heterozygous state with the mild c.245C>T (p.Thr82Met) variant in a patient with MBD plus^30^. As was shown earlier c.245C>T is the splicing variant, which is located outside of the canonical dinucleotide and could potentially lead to some residual amount of WT mRNA isoforms^31^. There is also another non-canonical splicing variant c.246G>T (p.Thr82Thr) identified in the MBD patient^32^. Thus, the potential “leakiness” of these noncanonical splicing variants could be the reason for their hypomorphic effect and association mainly with MBD, rather than GM1 gangliosidosis phenotype.

## Conclusion

To our knowledge, this is the second reported case of the processed pseudogene insertion for monogenic disorder. We demonstrated the rare type of molecular genetic mechanism of pathogenesis, which involves an alteration of splicing regulatory elements’ landscape and utility of minigene assay and ASMOs in functional analysis and correction of such types of genetic variants. The results of our work emphasize the thorough analysis of NGS data, as it allowed us not only to detect the footprints of the retrotransposition, but also to identify the separate genetic cause of the patient’s hearing loss.

## Methods

The study was approved by the local ethics committee of the Federal State Budgetary Institution “Research Centre for Medical Genetics” (the approval number 2015-5/3). The written informed consent was obtained from the patients’ parents and the protocol was approved by the local Institutional Review Board.

### Biochemical analysis

The activity of lysosomal enzymes was measured in dried blood spot samples by LC-MS/MS method. The internal standards and substrates for *GLB1, I2S, NAGLU, GALNS, ARSB, GUSB*, and *TPP1* were commercially purchased from PerkinElmer, Inc. (Waltham, MA, USA).

The multiplex assay was performed as follows. A 3-mm punch of dried blood spot was incubated in the buffer containing four substrates and internal standards overnight. A liquid–liquid extraction by using aqueous NaCl and ethyl acetate was performed. Subsequently, the ethyl acetate layer was then collected and dried. The sediment was consequentially resuspended in solvent for auto-sampling for tandem mass spectrometry analysis.

Samples were measured using a LC-30 Nexera System (Shimadzu Corporation, Kyoto, Japan) and a tandem mass spectrometer QTrap 4500 (ABSciex, USA) equipped with an positive electrospray ionization. The LC column was a Phenomenex Fusion-RP 50 × 2.1 mm, 4 µm (Phenomenex, Torrance, CA, USA), and the column oven temperature was 50 °C.

### DNA analysis

Genomic DNA was extracted from whole blood with EDTA using GeneJET Genomic DNA Purification Kit (Thermo Fisher Scientific, Waltham, MA, USA). Sanger sequencing was performed on ABI PRISM 3500xL Genetic Analyzer (Thermo Fisher Scientific, Waltham, MA, USA). Whole-genome sequencing of the patient’s DNA was performed with TruSeq DNA PCR-Free sample preparation kit on NovaSeq 6000 (Illumina, San Diego, CA, USA) with mean coverage of 42X.

Bioinformatics pipeline: sequence reads were aligned to the human reference genome GRCh37 (hg19) using Burrows-Wheeler Aligner v.0.7.17-r1188 (http://bio-bwa.sourceforge.net, accessed on 13th Jun 2022). Single-nucleotide variants and small insertions and deletions (indels) were called with Strelka2 Small Variant Caller v.2.9.10 (https://github.com/Illumina/strelka, accessed on 13th Jun 2022) and the Genome Analysis Toolkit v.4 (https://gatk.broadinstitute.org, accessed on 13th Jun 2022). Structural variants were called with Manta v. 1.6.0 (https://github.com/Illumina/manta, accessed on 13th Jun 2022). The reported variants were annotated with their genomic coordinates, allele frequency (gnomAD database, http://gnomad.broadinstitute.org, accessed on 13th Jun 2022), functional consequence, and impact level on the gene product using SnpEff v5 (http://pcingola.github.io/SnpEff, accessed on 13th Jun 2022). Variants were prioritized by the consensus score of the set of bioinformatic tools, which predict the pathogenicity of the variant and the deleterious effect on protein (SIFT, SIFT4G, Polyphen2, MutationAssessor, FATHMM, PROVEAN, DEOGEN2, LRT, PrimateAI, MetaSVM, MetaLR, SpliceAI, MMsplice, SPiP, Spidex). Data analysis was performed with custom web-based NGS-data-Genome interface.

Variants were named according to the *GLB1* reference sequence NM_000404.4 and GRCh37.p13 (hg19) genome assembly.

### RNA analysis

The patient’s total RNA was isolated from cultured fibroblasts using Total RNA Purification Plus Kit (Norgene, Thorold, ON, Canada). The first strand of cDNA was synthesized using ImProm-II™ Reverse Transcriptase (Promega, Madison, WI, USA) and oligo(dT) primers. Overlapping fragments of the *GLB1* cDNA were amplified by PCR and Sanger sequenced.

### Co-transfection of minigenes and antisense splice modulating oligonucleotides

The 505 bp fragment of the (WT) *GLB1* intron 5 (NC_000003.11:g.33099863_33100367) and the ~1850 bp fragment with PP insertion were placed between two constitutively spliced exons (V1 and V2) of the pSpl3-Flu2-mTK vector. pSpl3-Flu2-mTK is a modification of the pSpl3-Flu vector^33^, in which the CMV promoter was changed to the miniTK promoter (the −33 to +32 region of the Herpes simplex thymidine kinase promoter) and the strong cryptic splice site downstream of the multiple cloning site was deleted. These modifications were made as they improved the recognition of a number of previously studied exons (unpublished data).

The 425 bp fragment of the mouse U7-snRNA gene containing promoter and terminator sequences was amplified with tailed primers 5′-ttaaAGATCTtaacaacataggagctgtg-3′ and 5′-ttaaCTCGAGcacatacgcgtttcctagg-3′ and cloned into pcDNA3.1 vector between BglII and XhoI restriction sites. Overlap-extension PCR was used to introduce several modifications. At first, U7-specific Sm binding site (AATTTGTCTAG) was replaced by the consensus Sm binding site (AATTTTTGGAG), thus incorporating the modified snRNA into the snRNP complex targeting the spliceosome^34^. For a number of constructs, the sequence, containing heterogeneous ribonucleoprotein A1 (hnRNPA1) binding sites (ATGATAGGGACTTAGGGTG) was added at the 5′ end of the coding sequence to improve the efficiency of splicing inhibition^35^. The 18 bp sequence, which is complementary to the histone pre-mRNA (AAGTGTTACAGCTCTTTT) is replaced by various antisense sequences, targeting the studied pre-mRNA region.

Identification of splicing regulatory motifs was performed with HExoSplice^36^ (http://bioinfo.univ-rouen.fr/HExoSplice_submit/inputs.php, accessed on 13th Jun 2022). Scores of splice sites were calculated by MaxEntScan (http://hollywood.mit.edu/burgelab/maxent/Xmaxentscan_scoreseq.html, accessed on 13th Jun 2022).

Plasmids with modified U7-snRNA genes were co-transfected with minigenes (250 ng of both plasmids in 24-well plate cell) into HEK293T cells (ATCC CRL-3216™) using Lipofectamine 3000 reagent (Thermo Fisher Scientific, Waltham, MA, USA). After 48 h, cells were harvested for RNA isolation and reverse transcription. Minigene-specific primers with 6-FAM modification located in the exons V1 and V2 were used to amplify the splicing products, which were further visualized by polyacrylamide gel electrophoresis and quantitatively analyzed by fragment analysis. Fragment analysis was performed using ABI PRISM 3500xL Genetic Analyzer (Thermo Fisher Scientific, Waltham, MA, USA) and Coffalyser.Net software v.220513.1739 (https://support.mrcholland.com/downloads/coffalyser-net, accessed on 13th Jun 2022).

Sequences of primers used in this study are listed in Supplementary Note 3.

### Reporting summary

Further information on research design is available in the Nature Research Reporting Summary linked to this article.

## Supplementary information

Supplementary material

Reporting Summary

## Data Availability

Data which support the findings of this study are available from the corresponding author upon request. The identified variants were deposited in the ClinVar database (https://www.ncbi.nlm.nih.gov/clinvar/). Accession IDs: VCV001344904.1, VCV001687042.1, VCV001687043.1 (accessed on 13th Jun 2022). Exome and genome sequencing data are not publicly available due to privacy and patient anonymity issues. This data are available from the corresponding author upon request and in accordance with the Data Usage Agreement.

## Code availability

The next-generation sequencing data were processed using freely available code described in the Methods. The custom web-based NGS-data-Genome interface was used to visualize and filter the resulting data and its code is available from the corresponding author upon reasonable request.
