# Publisher Correction: Processed pseudogene insertion in *GLB1* causes Morquio B disease by altering intronic splicing regulatory landscape

**DOI:** 10.1038/s41525-022-00337-6

**Published:** 2022-11-14

**Authors:** Igor Bychkov, Antonina Kuznetsova, Galina Baydakova, Leonid Gorobets, Vladimir Kenis, Alena Dimitrieva, Alexandra Filatova, Vyacheslav Tabakov, Mikhail Skoblov, Ekaterina Zakharova

**Affiliations:** 1 Research Centre for Medical Genetics, Moscow, Russia; 2 Clinical and Diagnostic Center “Zdorovoe detstvo”, Rostov-on-Don, Russia; 3 H. Turner National Medical Research Centre for Children’s Orthopedics and Trauma Surgery, Saint Petersburg, Russia

**Keywords:** Metabolic disorders, Mobile elements, RNA splicing

Correction to: *npj Genomic Medicine* 10.1038/s41525-022-00315-y, published online 26 July 2022

The original version of this article contained an error in the final sentence of the second paragraph of the “Co-transfection of minigenes and antisense splice modulating oligonucleotide” section of the Methods. The text incorrectly read “Sequences of primers used for creating modified U7-snRNA”. The correct sentence at the end of the section reads “Sequences of primers used in this study are listed in Supplementary Note 3”. Also, in the second sentence of the Methods section “Co-transfection of minigenes and antisense splice modulating oligonucleotides”, the miniTK promoter was wrongly referred to as Minick. These corrections have been made in both the PDF and HTML versions of the article.

