# Supplementary material for: Processed pseudogene insertion in GLB1 causes Morquio B disease by altering intronic splicing regulatory landscape
Source: NPJ Genom Med. 2022 Jul 26;7:44. doi: 10.1038/s41525-022-00315-y (PMC9325892; doi:10.1038/s41525-022-00315-y)
Supplement: Supplementary file 1 — Supplementary material [file 41525_2022_315_MOESM1_ESM.docx]

Supplementary materials for

**Processed pseudogene insertion deep in the intron of *GLB1* causes Morquio B disease by altering splicing regulatory landscape**

Igor Bychkov^1^, Antonina Kuznetsova^1^, Galina Baydakova^1^, Leonid Gorobets^2^, Vladimir Kenis^3^, Alyona Dimitrieva^3^, Alexandra Filatova^1^, Vyacheslav Tabakov^1^, Mikhail Skoblov^1^, Ekaterina Zakharova^1^

^1^ Research Centre for Medical Genetics, Moscow, Russia

^2^ Clinical and diagnostic center “Zdorovoe detstvo”, Rostov-on-Don, Russia

^3^ H. Turner National Medical Research Centre for Children’s Orthopedics and Trauma Surgery, Saint Petersburg, Russia

Correspondence: Bychkov I.O. (bychkov@med-gen.ru)

**Supplementary Note 1. Analysis of the identified *CDH23* gene variants**

The additional findings from the whole exome data analysis include two rare missense variants in the *CDH23* gene (NM_022124.6): c.805C>T (p.Arg269Trp) and c.6992T>C (p.Val2331Ala). This gene encodes calcium-dependent cell adhesion glycoprotein cadherin-23, which is involved in a proper organization of cochlear, vestibular and retinal cells. Depending on the severity of the genetic defect, pathogenic variants in *CDH23* are associated with two main phenotypes. Biallelic nonsense, frameshift, splice site and severe missense variants are associated with Usher syndrome, type 1D (MIM: 601067), characterized by deafness, vestibular areflexia, and retinitis pigmentosa [1]. Hypomorphic missense variants in homozygous or compound heterozygous state preserve the visual and vestibular functions and lead to non-syndromic deafness (MIM: 601386). c.805C>T and c.6992T>C variants were not described previously and have low allele frequency. The segregation analysis confirmed the compound-heterozygous state of the variants in patient AI and his brother DI with non-syndromic deafness (Fig. 1d). According to the ACMG classification implemented into Varsome V 11.2.8 (https://varsome.com/, web interface accessed on 13th Jun 2022), ClinGen Sequence Variant Interpretation Recommendations (accessed on 13th Jun 2022) and data acquired during this study, c.805C>T and c.6992T>C variants can be classified as likely pathogenic variants (c.805C>T: PM1 moderate, PM2 supporting, PM3 supporting, PP1 supporting, PP3 supporting; c.6992T>C: PM2 strong, PM3 supporting, PP1 supporting). The c.6992T>C variant is predicted to have small or no deleterious effect on protein structure and function. The PredictSNP consensus score (https://loschmidt.chemi.muni.cz/predictsnp/, web interface accessed on 13th Jun 2022) is 55% deleterious for c.805C>T and 63% benign for c.6992T>C. The Varsome pathogenicity predictions are 6 benign and 17 pathogenic scores for c.805C>T and 12 benign, 10 pathogenic and 1 neutral score for c.6992T>C. This data corresponds well with the necessity of the presence of at least one hypomorphic variant in *CDH23* for isolated hearing loss in our patients.

**Supplementary Note 2. The prediction of the in-frame insertion (p.Gln184_Val185insHFYHGK) impact on the *GLB1* protein function**

1. MutPredIndel: a predictor of impactful non-frameshifting indel variants (mutdb.org, web interface accessed on 13th Jun 2022).

MutPred-Indel score: 0.85879 (>0.85 – highly pathogenic). Affected features (p<0.05 – significant):    Magnesium binding (p=0.014507); Cadmium binding (p=0.018964); Manganese binding (p=0.01922); Zinc binding (p=0.030815); Allosteric site (p=0.0368).

1. PROVEAN (Protein Variation Effect Analyzer) is a tool which predicts whether an amino acid substitution or indel has an impact on the biological function of a protein (http://provean.jcvi.org/index.php, web interface accessed on 13th Jun 2022).

PROVEAN score: -13.336 (<-2.5 Deleterious).


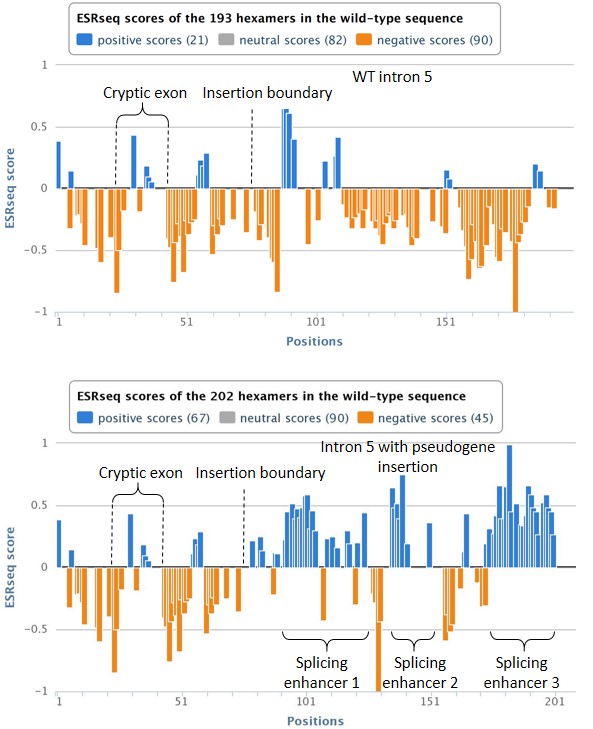


**Supplementary Figure 1.** The HExoSplice analysis of the *GLB1* intron 5 fragment (chr3:33,099,929-33,100,126) containing the cryptic exon. The wild type fragment is enriched with motifs, inhibiting the recognition of the cryptic exon (splicing silencers, orange bars), while the PP-derived sequence is enriched with the motifs which promote its recognition (splicing enhancers, blue bars). Three clusters of splicing enhancer motifs were called Splicing enhancer 1-3 and correspond to E1-3 in Fig. 2 c,d in the main text.


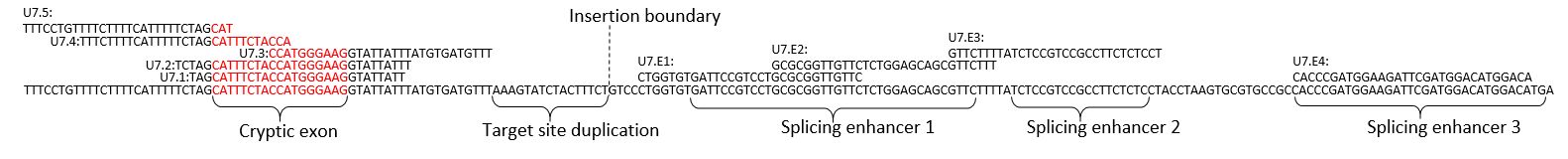


**Supplementary Figure 2**. The *GLB1* intron 5 fragment with PP insertion at the sequence level and location of ASMOs.

**Supplementary Note 3. Primers and sequences**

| modU7snRNA | Target on the sense strand | Anitsense sequence | Length |
| --- | --- | --- | --- |
| U7.1 | tagcatttctaccatgggaaggtattatt | aataataccttcccatggtagaaatgcta | 29 |
| U7.2 | tctagcatttctaccatgggaaggtattattt | aaataataccttcccatggtagaaatgctaga | 32 |
| U7.3 | ccatgggaaggtattatttatgtgatgttt | aaacatcacataaataataccttcccatgg | 30 |
| U7.4 | tttcttttcatttttctagcatttctacca | tggtagaaatgctagaaaaatgaaaagaaa | 30 |
| U7.5 | tttcctgttttcttttcatttttctagcat | atgctagaaaaatgaaaagaaaacaggaaa | 30 |
| U7.E1 | ctggtgtgattccgtcctgcgcggttgttc | gaacaaccgcgcaggacggaatcacaccag | 30 |
| U7.E2 | gcgcggttgttctctggagcagcgttcttt | aaagaacgctgctccagagaacaaccgcgc | 30 |
| U7.E3 | gttcttttatctccgtccgccttctctcct | aggagagaaggcggacggagataaaagaac | 30 |
| U7.E4 | cacccgatggaagattcgatggacatggaca | tgtccatgtccatcgaatcttccatcgggtg | 31 |

| Purpose/target | Forward primer | Reverse primer |
| --- | --- | --- |
| Amplification of the processed pseudogene insertion in the GLB1 intron 5 | 5’-gtctgtgtaaatctagacggctgt | 5’-ctaatgcaagcttttgtgtggg |
| *CDH23* exon 9 | 5’-atctgttcttccgtggtggtc | 5’-ctcagaccttggacagcgg |
| *CDH23* exon 50 | 5’-ggtcaggtcaatcctcaggc | 5’-cctccttcttggccttcctg |
| *GLB1* exon 8 | 5’-ggcattggaaggtcataagtc | 5’-gaacacggtacttcactgtga |
| *GLB1* cdna fragment 1 | 5’-tttcctgttttcttttcatttttctagcat | 5’-atgctagaaaaatgaaaagaaaacaggaaa |
| *GLB1* cdna fragment 2 | 5’-ctggtgtgattccgtcctgcgcggttgttc | 5’-gaacaaccgcgcaggacggaatcacaccag |
| *GLB1* cdna fragment 3 | 5’-gcgcggttgttctctggagcagcgttcttt | 5’-aaagaacgctgctccagagaacaaccgcgc |
| *GLB1* cdna fragment 4 | 5’-gttcttttatctccgtccgccttctctcct | 5’-aggagagaaggcggacggagataaaagaac |
| Amplification of the exon 4 and 5 junction | 5’-tctgcccaagatgaagcctc | 5’-ctccatcagtggtaaacagaacc |
| Cloning of the processed pseudogene insertion in the expression vector | 5’-aattCTCGAGacatgctgtgtctgtccctg | 5’-aattGGATCCcaaggcttaatgactgctggaa |

Primers for introducing antisense sequences into mU7-snRNA cassette are available upon request.


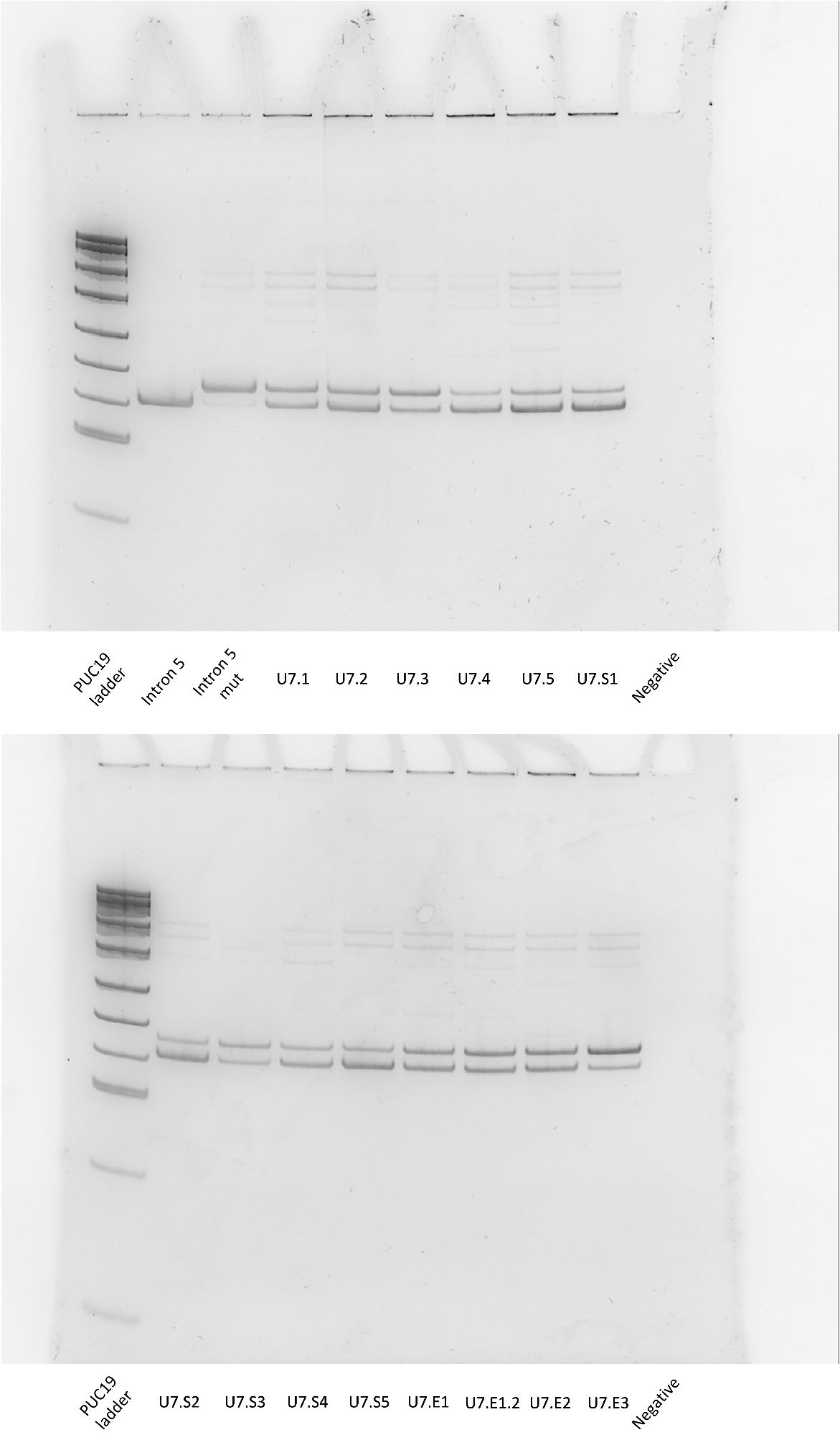


**Supplementary Figure 3.** Un-cropped blots for Figure 2.

**Supplementary References**

1. Schultz, J.M., et al., *Allelic hierarchy of CDH23 mutations causing non-syndromic deafness DFNB12 or Usher syndrome USH1D in compound heterozygotes.* Journal of medical genetics, 2011. **48**(11): p. 767-775.
